# Physiologically-based pharmacokinetic model to predict pharmacokinetics of KY0467 in humans and guide dose selection in infants and children

**DOI:** 10.3389/fphar.2026.1866331

**Published:** 2026-10-12

**Authors:** Panpan Xie, Yanyan Yang, Xuemei He, Ming Yang, Zhixing Chen, Xi Weng, Aixin Shi

**Affiliations:** Clinical Trial Center, Beijing Hospital, National Center for Gerontology, National Clinical Research Center for Gerontology, The Key Laboratory of Geriatrics of NHC, Institute of Geriatric Medicine, Chinese Academy of Medical Sciences, Beijing Key Laboratory of Geriatrics-Based and AI-Driven Pharmaceutical and Medical Device Evaluation and Translation, Beijing, China

**Keywords:** children, HFMD, infants, KY0467, pediatric dose selection, pharmacokinetics, physiologically based pharmacokinetic (PBPK) modeling

## Abstract

**Background:**

Hand, foot and mouth disease (HFMD) caused by enterovirus 71 (EV71) remains a severe threat to infants and young children, with no specific antiviral therapy currently available. KY0467 is a novel EV71‐targeting antiviral drug under clinical development, but clinical data are limited to healthy adults, and appropriate pediatric dosing regimens are urgently needed.

**Methods:**

In this study, a physiologically based pharmacokinetic (PBPK) model was developed and validated in rats and beagles, extrapolated to humans, and verified using clinical pharmacokinetic (PK) data from healthy adults. The model was then scaled to infants (28 days–23 months) and children (2–5 years) using age‐dependent physiological parameters to predict effective doses matching adult exposure at 30 mg/day.

**Results:**

Predicted plasma concentration‐time profiles agreed well with observed values, and all mean fold errors (MFE) for T_max_, C_max_, and AUC_0‐t_ were within 0.5‐ to 2‐fold. Population simulations identified effective doses of 0.5 mg/kg/day for infants and 9 mg/day for children.

**Conclusions:**

In conclusion, the adult PBPK model of KY0467 was successfully developed and validated for the first time, and the extrapolated pediatric model could be used to predict pediatric dosing of KY0467, which will provide invaluable references for the design of future clinical trials and its rational use in the pediatric population.

## 1 Introduction

Hand, foot, and mouth disease (HFMD) is a common infectious disease that predominantly affects children under 5 years of age. Enterovirus 71 (EV71) and coxsackievirus A16 (CVA16) are the primary causative pathogens. Severe cases may develop serious complications including central nervous system disorders, respiratory distress, cardiovascular dysfunction, and even death, with EV71 accounting for approximately 90% of fatal HFMD cases (Zhang M et al., 2020; Nayak G et al., 2022; Qing J et al., 2014). Since 1997, outbreaks of hand, foot, and mouth disease (HFMD) have occurred throughout the Asia-Pacific region. In 2010, mainland China reported more than one million infections and over nine hundred deaths attributable to HFMD. Consequently, HFMD has emerged as a pressing public health concern worldwide (Leung et al., 2022; Lim ZQ et al., 2019; Cui Y et al., 2022). Although an EV71 vaccine has been widely used, it does not provide therapeutic effects for infected children. Therefore, effective antiviral drugs targeting EV71 are urgently needed.

KY0467 is a novel antiviral agent that specifically targets EV71 by binding to the VP1 protein hydrophobic pocket, thereby inhibiting viral RNA release and replication. Preclinical studies have demonstrated favorable antiviral activity and pharmacokinetic (PK) properties in rats and beagles. The pharmacokinetic (PK) parameters of KY0467 in rats and beagles, including clearance, volume of distribution, and half-life, are summarized in Supplementary Table S1. These findings provide a solid foundation for the clinical translation of KY0467.

A Phase I clinical study was conducted in healthy adults, but the target population is infants and children. Due to significant developmental differences in physiology and metabolism, simple allometric scaling cannot accurately predict pediatric PK. Physiologically based pharmacokinetic (PBPK) modeling is essential, as it mechanistically integrates age-dependent physiological changes (e.g., organ maturation and enzyme ontogeny) with drug-specific properties. This approach enables the virtual simulation of drug exposure in children, minimizing the need for extensive and ethically challenging clinical trials in pediatric populations. It provides a scientifically rigorous, evidence-based framework for first-in-pediatric dose selection and supports the rational design and ethical justification of necessary clinical studies.

PBPK modeling and simulation is an *in silico* approach that integrates anatomical and physiological parameters-such as blood flow and tissue composition-to characterize the pharmacokinetic (PK) profiles of drugs (Zhuang and Lu, 2016). PBPK models also account for age-related variations in physiological parameters, enabling simulation of age-dependent changes in drug absorption, distribution, and metabolism. In recent years, PBPK models have been increasingly applied across various stages of drug development and are particularly valuable for predicting drug behavior in special populations, including pediatric patients.

In pediatric clinical research, PBPK modeling has been used to identify initial doses, effective doses, and maximum tolerated doses. Jo et al. developed and verified a dapagliflozin PBPK model incorporating UGT1A9 ontogeny to guide pediatric dose selection, successfully determining absolute oral doses for pediatric patients (1 month–18 years of age) that closely matched exposure following a 10 mg adult dose (Jo H et al., 2021). Liu et al. established a PBPK model for lamotrigine to predict optimal pediatric dosing regimens and successfully guided dose adjustments in children aged 2–12 years receiving combination therapy with carbamazepine or valproic acid (Liu Z et al., 2024).

The present study aimed to a) develop and validate a PBPK model of KY0467 in rats and beagles validated by observed data, b) extrapolate the model to predict pharmacokinetic (PK) profiles in humans, with further validation using observed clinical datas, c) ultimately extrapolate the model to infants (28 days-23 months) and children (2–5 years), to explore effective doses for these populations to match adult target exposure.

## 2 Materials and methods

### 2.1 Software and data sources

A full PBPK model of KY0467 was developed using GastroPlus™ (version 9.9; Simulations Plus, Inc., Lancaster, CA, USA). Pharmacokinetic profiles were generated using Origin 2022 software. The physicochemical and ADME properties used in the PBPK model are summarized in Table 1. The experimental design and technical roadmap of this study are presented in Figure 1.

**TABLE 1:** Physicochemical and ADME parameters required for PBPK model development.

Parameter	Value	Source
Molecular weight (g/moL)	538.07	measured data
Log P	3.6	measured data
pKa	6.7	measured data
Solubility (μM)	183 (pH = 2) <1 (pH = 7&9)	measured data
Particle size (D_50_) (μm)	4.4	measured data
Caco-2 permeability (10^−5^ cm/s)	0.38	measured data
B/P	1.1 (Rat, Beagle, human)	Predicted
F_u,p_	2.4 (Rat), 1.1 (Beagle), 1.83 (Human)	measured data
CL_int,_ _*in vitro*_ (mL/min/kg)	34.5	measured data
t_1/2_ (min)	39.8	measured data
CYP3A4 K_m_,unbound (μM)	0.031	Data was optimized based on observed human non-linear PK
CYP3A4 V_max_, (pmol/min/pmolCYP3A4)	0.104	Data was optimized based on observed human non-linear PK

logP, partition coefficient; pKa, dissociation constant; B/P, blood to plasma concentration ratio; fu,p, unbound fraction in plasma; CL_int, *in vitro*,_ *In vitro* intrinsic clearance rate; t_1/2,_half-life time.

**FIGURE 1:**
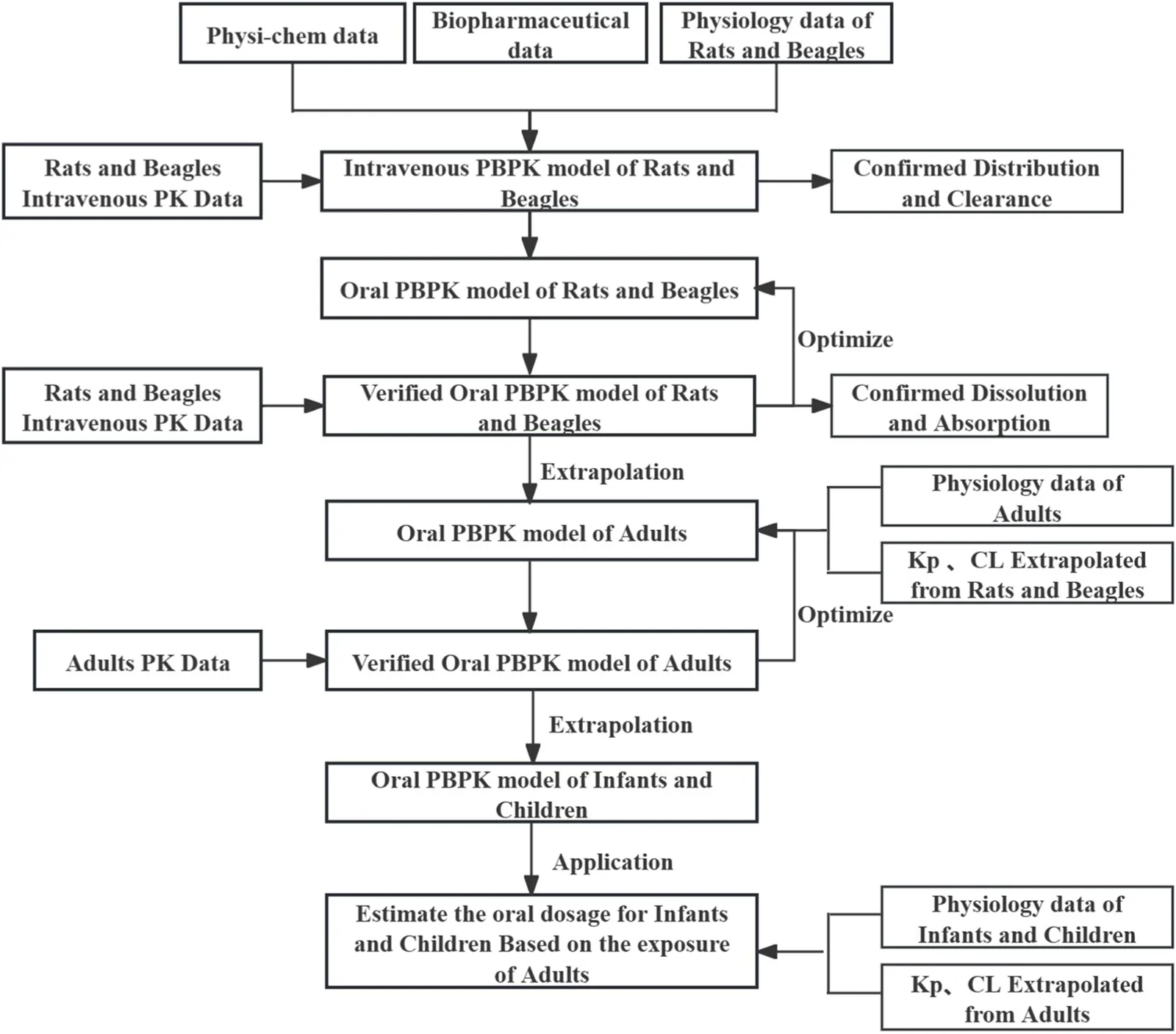
The experimental design and technical roadmap of this study.

### 2.2 Preclinical study design

Rats (n = 6 per group) were administered KY0467 intravenously at a dose of 2.0 mg/kg and orally at doses of 15, 50, and 150 mg/kg. Blood samples were collected at pre-dose (0 h), 0.083, 0.25, 0.5, 1, 2, and 4 h after intravenous administration; and pre-dose (0 h), 0.25, 0.5, 1, 2, 4, 8, 12, and 24 h after oral administration. Blood samples were collected into K_2_-EDTA tubes and immediately centrifuged at 3000 *g* for 15 min at 4 °C. The separated plasma was stored at a temperature −60 °C or lower until analysis.

Beagles (n = 6 per group) received KY0467 intravenously at 1.0 mg/kg and orally at 10, 30, and 90 mg/kg. Blood samples were collected at pre-dose (0 h), 0.083, 0.25, 0.5, 1, 2, 4, 6, 8, and 12 h after intravenous dosing; and pre-dose (0 h), 0.25, 0.5, 1, 2, 3, 4, 6, 8, 12, and 24 h following oral administration. Blood samples were collected into K_2_-EDTA·2H_2_O tubes and immediately centrifuged at 3000 *g* for 10 min at 4 °C. The separated plasma was stored at a temperature −60 °C or lower until analysis.

### 2.3 Clinical study design

This was a randomized, double-blind, placebo-controlled, single-ascending dose study conducted at Beijing Hospital. The study was approved by the Institutional Ethics Committee of Beijing Hospital (Approval No.:2019BJYYEC-123–01) and conducted in accordance with the Declaration of Helsinki and Good Clinical Practice (GCP) guidelines. All participants provided written informed consent. Regarding demographics, there were no female participants in the 10 mg (n = 3) and 30 mg (n = 8) groups (0%), while the 100 mg group (n = 8) included two females (25.0%). The age ranges (minimum–maximum) for the 10 mg, 30 mg, and 100 mg groups were 21–35 years, 25–40 years, and 26–45 years, respectively; corresponding body weight ranges were 61.5–73.0 kg, 58.6–81.3 kg, and 52.4–84.5 kg.

Following single-dose oral administration of 10 mg, 30 mg, and 100 mg of KY0467 as an oral solution, samples were collected at pre-dose (within 1 h prior to dosing), 0.25, 0.5, 1, 2, 4, 6, 8, 12, 16, 24, 48, and 72 h post-administration. The concentrations of KY0467 in plasma were determined using a validated liquid chromatography-tandem mass spectrometry (LC-MS/MS) method. The calibration curve showed good linearity over the range of 0.5–250 ng/mL.This trial is registered with the Chinese Clinical Trial Registry (registration number: ChiCTR2400081388).

### 2.4 Animal PBPK model development and verification

The PBPK model of KY0467 was initially developed and validated in rats and beagles by comparing simulated plasma exposure with observed data. Absorption parameters were estimated using the Advanced Compartmental Absorption & Transit (ACAT) model and Johnson model. Species-specific physiological parameters were obtained from the default parameters built into the software. Tissue distribution was assumed to be perfusion-limited. The volume of distribution was predicted using the Lukacova method (Yim and Choi, 2020; Jones RD et al., 2011; Holt K et al., 2019). Clearance was scaled based on *in vivo* intravenous data.

### 2.5 Adult PBPK model development and verification

The validated animal PBPK model was extrapolated to humans using the same absorption and tissue distribution models. Age-specific physiological parameters were derived from pre-built age-dependent algorithms in PEAR Physiology™ (Population Estimates for Age-Related Physiology). Multiple approaches were employed to predict human clearance. An *in vitro* to *in vivo* extrapolation (IVIVE) method was applied: *in vitro* intrinsic clearance (CL_int, *in vitro*_) was calculated based on the elimination half-life of KY0467,and human *in vivo* intrinsic clearance (CL_int_) was then determined using physiological scaling factors (such as hepatic microsomal protein concentration and liver weight). Hepatic *in vivo* clearance (CL_H_) was subsequently estimated using the well-stirred liver model with hepatic blood flow, ignoring plasma protein binding effects (Tang C et al., 2021; Gao ZW et al., 2015; Barter ZE et al., 2007). Several allometric scaling methods were also used to predict human clearance, including single-species allometric scaling (SSS_rat_, SSS_beagle_), two-species allometry scaling (TS_rat-beagle_), and the fraction unbound intercept correction method (FCIM) (Ring BJ et al., 2011; Goteti K et al., 2010; Espié P et al., 2009).

### 2.6 Model extrapolation to pediatric

For the development of pediatric PBPK models, the validated adult PBPK model was scaled to pediatric populations. All drug-specific parameters (e.g., physicochemical properties) were kept fixed. To account for age-dependent physiological differences, pediatric populations were established using the built-in PEAR Physiology™ module in GastroPlus. This module incorporates adjusted anatomical and physiological parameters-including body weight, height, organ volume, tissue blood flow, hematocrit, total body water, lipid content, and protein concentrations-based on mean values reported for the Chinese pediatric population. Representatives ages (0, 0.5, 1, 2.5, 5, 10, and 15 years) from the module were selected to correspond to age classifications defined in the ICH guideline: neonates (0–27 days), infants (28 days - 23 months), children (2–11 years), and adolescents (12–17 years) (Shao W et al., 2023). The ontogeny profiles for CYP3A4 built into GastroPlus were used to characterize postnatal changes in metabolic clearance, reflecting developmental changes in enzyme expression levels in both the liver and intestine.

The mean unbound plasma drug fraction (fup) and whole blood-to-plasma drug concentration ratio (Rbp) were differedbetween pediatric and adult populations. The fup in pediatric was calculated using the McNamara and Alcorn equation as follows (McNamara and Alcorn, 2002):

$${Fup}_{ped}=\frac{1}{1+\frac{P_{ped}}{P_{adult}}\times \frac{\left(1-{Fup}_{adult}\right)}{{Fup}_{adult}}}$$ (1)

Where *P*_*ped*_ refers plasma protein concentration in pediatric population; *P*_*adult*_ refers plasma protein concentration in adults; *F*_*up,ped*_ refers to the unbound plasma drug fraction in pediatric population; *F*_*up,adult*_ refers the unbound plasma drug fraction in adults. The pediatric to adult plasma protein ratio (*P*_*ped*_ */P*_*adult*_) was derived based on the ontogeny profiles of the two major drug-binding proteins in plasma: α_1_--acid glycoprotein (AAG) and albumin.

The whole blood-plasma drug distribution ratio were calculated using the equation in pediatric as follows:

$${Rbp}_{ped}=\frac{{Hct}_{ped}}{{Hct}_{adult}}\times \left({Rbp}_{adult}-\left(1-{Hct}_{adult}\right)\right)+\left(1-{Hct}_{ped}\right)$$ (2)

Hct_ped_ and Hct_adult_ represent hematocrit (expressed as a fraction) in pediatric and adult blood, respectively, Hct_adult_ was set to 0.45. Rbp_ped_ and Rbp_adult_ represent blood to plasma concentration ratio in pediatric and adult blood, respectively.

### 2.7 PBPK model evaluation

PBPK models were evaluated by visual predictive checks comparing simulated and observed concentration–time profiles, In addition, as quantitative metrics, the area under the plasma concentration-time curve (AUC_0-t_), maximum plasma concentration (C_max_), and time to reach C_max_ (T_max_) were assessed, and the mean fold error (MFE) was calculated for these PK parameters as follows (Zheng L et al., 2019):

MFE = Predicted mean parameter/Observed mean parameter.

PBPK models were considered acceptable when all the predicted PK parameters were within two fold of the observed values.

## 3 Results

### 3.1 PBPK models validations in rats and beagles

The PBPK model of KY0467 was initially developed in rats and beagles following intravenous and oral administration. To avoid the complexity associated with absorption processes, intravenous data were utilized first for model establishment, which yielded distribution and elimination profiles consistent with observed values. Model performance evaluation was assessed by superimposing model-predicted with observed data. Predicted V_ss_ values were corresponded closely with observed data in both rats and beagles. Following intravenous administration, predicted AUC_0-t_ values showed minimal fold errors of 0.96 and 1.02 relative to observed data in rats and beagles, respectively. Furthermore, goodness-of-fit plots demonstrated stong agreement between predicted and observed plasma concentration-time profiles, and MFE values for AUC_0-t_, T_max_ and C_max_ all fell within the two fold prediction error range after oral administration of 15, 50 and 150 mg/kg in rats and 10, 30, and 90 mg/kg in beagles, as presented in Table 2 and Figures 2A–D.

**TABLE 2:** Predicted and observed pharmacokinetic parameters after oral KY0467 in rats and beagles.

Species	Dose (mg/kg)	C_max_ (ng/mL)			T_max_ (h)			AUC_0-t_ (ng · h/mL)		
		Pred	Obs	MFE	Pred	Obs	MFE	Pred	Obs	MFE
Rats	15	84.7	92.5	0.92	1.65	1	1.65	323.9	278.9	1.16
	50	369.5	357.3	1.03	1.56	1	1.56	1883.2	1551	1.21
	150	998.0	920.1	1.08	1.2	1	1.2	5936.8	6150	0.97
Beagles	10	624.5	565	1.11	1.52	2	0.76	3220.6	3772.4	0.85
	30	1938.4	2491.3	0.78	1.44	2	0.72	10570	12030	0.88
	90	1919.5	2173.8	0.88	2.32	4	0.58	14860	14610	1.02

**FIGURE 2:**
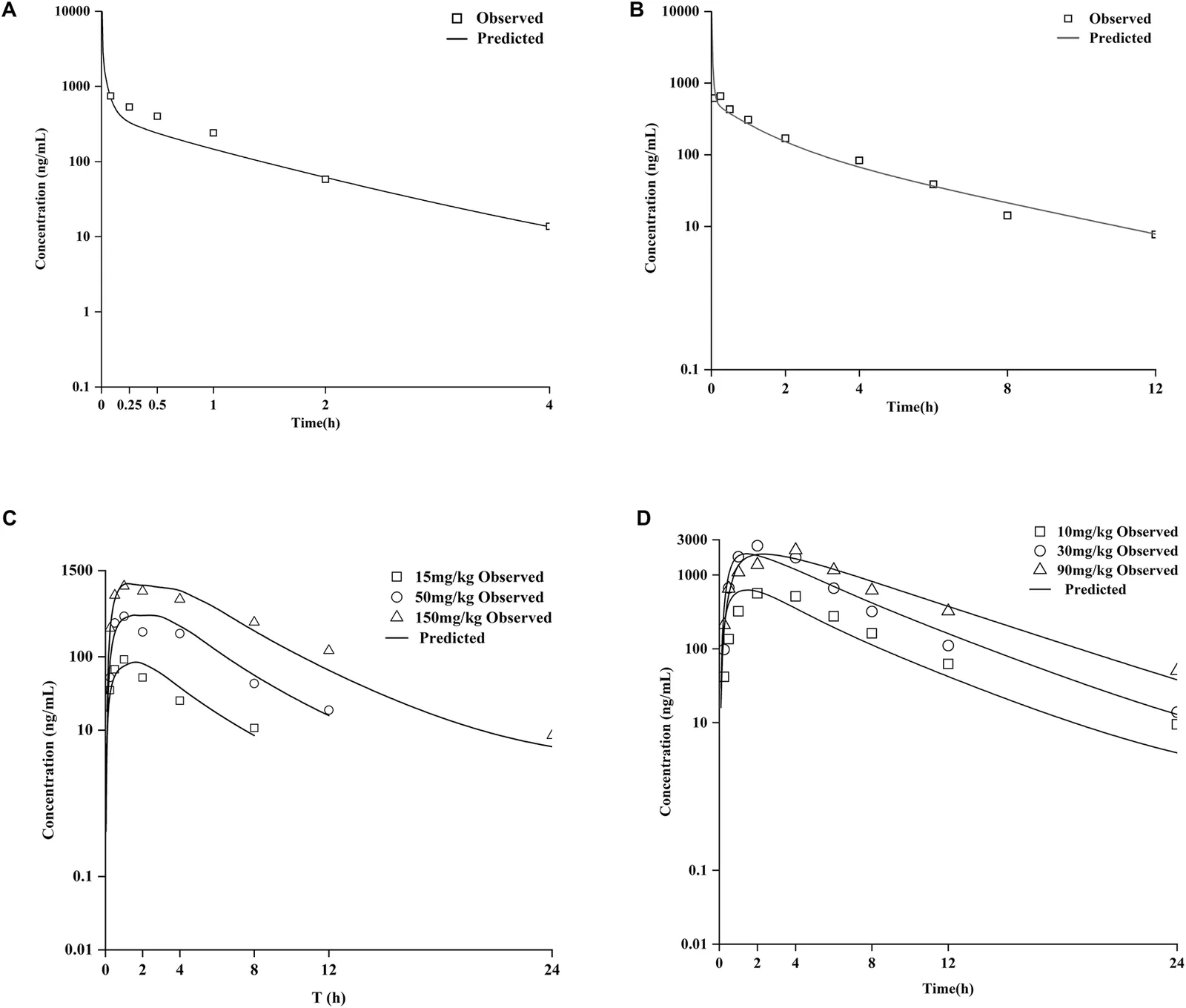
**(A)** Predicted and observed mean plasma concentration-time profiles of KY0467 after intravenous or oral administration in rats.Symbols represent observed data. Lines represent predicted data. **(B)** Predicted and observed mean plasma concentration-time profiles of KY0467 after intravenous in Beagles. Symbols represent observed data. Lines represent predicted data. **(C)** Predicted and observed mean plasma concentration-time profiles of KY0467 after oral administration in rats. Symbols represent observed data. Lines represent predicted data. **(D)** Predicted and observed mean plasma concentration-time profiles of KY0467 after oral administration in Beagles. Symbols represent observed data. Lines represent predicted data.

### 3.2 PBPK model validation in healthy adults

The same approach was applied to predict V_SS_ in humans. Human clearance (CL_human_) was predicted to be 41.75,10.70, 20.82, 47.63 and 14.07 L/h using the IVIVE, SSS_rat_, SSS_beagle_, TS_rat-beagle_ and FCIM methods, respectively. Among these, the value predicted by the TS_rat-beagle_ method showed the closest agreement with observed data following a 10 mg dose of KY0467, as shown in Figure 3A. The pharmacokinetics of KY0467 in humans exhibited nonlinear behavior over the dose range of 10–100 mg, attributable to saturation of hepatic CYP-mediated metabolism. CYP3A4 was identified as the major enzyme involved, responsible for approximately 90% of metabolic clearance, whereas urinary excretion played only a minor role in total elimination. Accordingly, the K_m_ and V_max_ parameters for CYP3A4 were optimized to reflect its dominant role in hepatic metabolism. Goodness-of-fit plots demonstrated strong agreement between predicted and observed plasma concentration-time profiles, and MFE values for AUC_0-t_, C_max_ and T_max_ all fell within the two fold prediction error range, as presented in Table 3 and Figure 3B.

**FIGURE 3:**
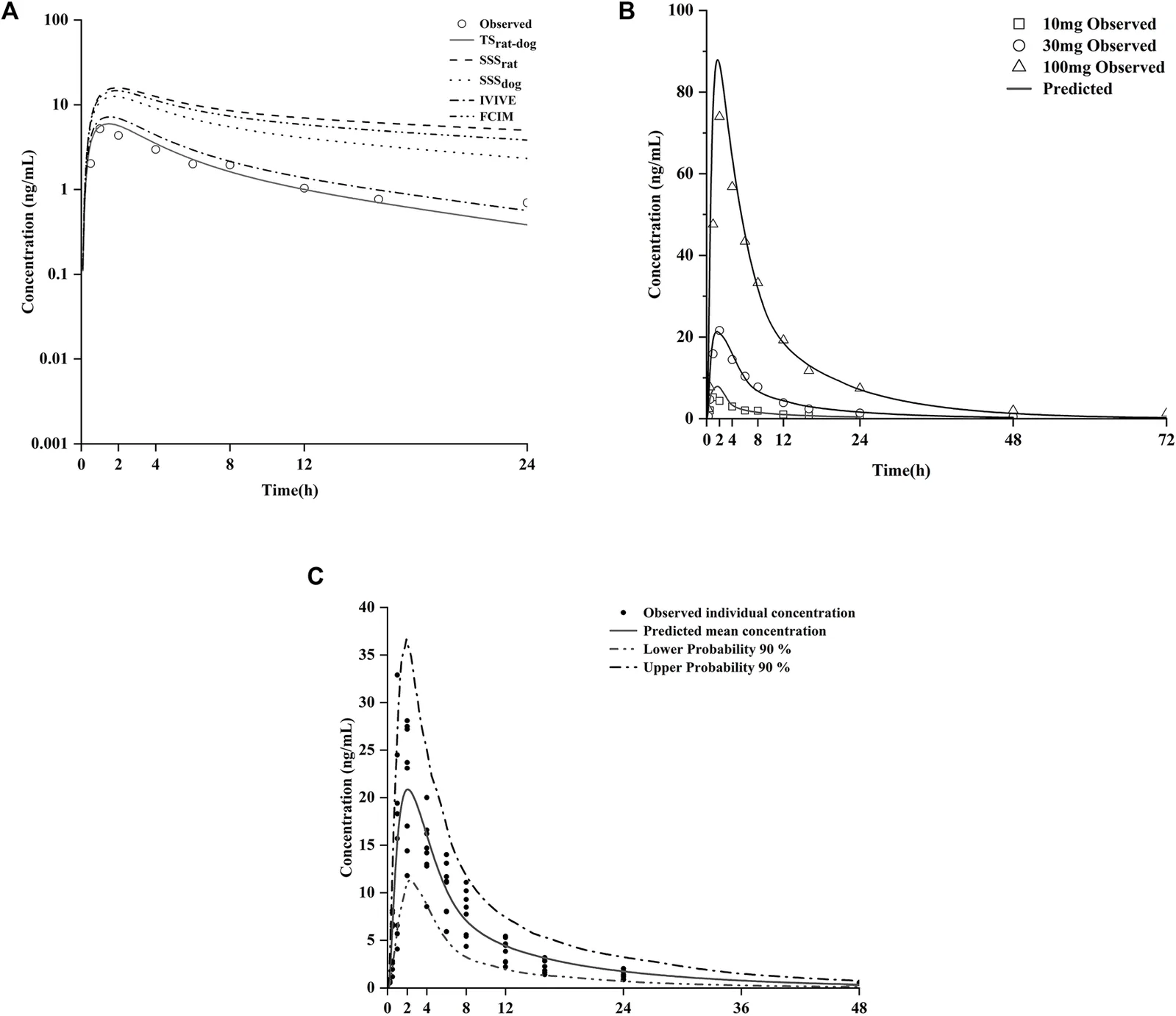
**(A)** Predicted and observed mean plasma concentration versus time profiles of KY0467 after oral administration of 10 mg KY0467 in healthy adults (n = 3) using IVIVE and allometric scaling methods. **(B)** Predicted and observed mean plasma concentration versus time profiles of KY0467 after oral administration of 10 mg (n = 3), 30 mg (n = 8) and 100 mg (n = 8) KY0467 in adults. **(C)** Individual plasma concentration in the virtual population of 30 mg (n = 8).

**TABLE 3:** Predicted and observed pharmacokinetic parameters after oral KY0467 in adults.

Dose (mg)	C_max_ (ng/mL)			T_max_ (h)			AUC_0-t_ (ng · h/mL)		
	Pred	Obs	MFE	Pred	Obs	MFE	Pred	Obs	MFE
10	7.92	5.22	1.52	1.68	1	1.68	44.60	38.92	1.15
30	21.32	21.6	0.99	1.6	2	0.8	183.5	178.3	1.03
100	87.94	73.98	1.19	1.68	2	0.84	797.9	854.2	0.93

The average plasma concentration of KY0467 required for effective antiviral activity was 9.6 ng/mL, with a target exposure (AUC) of approximately 230 ng h/mL. The effective dose in adults was estimated to range from 30 to 50 mg. The simulated population consisted of 100 virtual adults aged 25–40 years, with 50 males and 50 females per group. The simulation duration was 48 h. Following a 30 mg dose, approximately 100% of individual simulated data points fell within the 90% confidence interval, as shown in Figure 3C. These results demonstrated that the established adult PBPK model was robust and provided a reliable basis for extrapolation to special populations.

### 3.3 Pediatric PBPK model scaling

The validated adult PBPK model was extrapolated to predict the pharmacokinetics of KY0467 in infants and children across pediatric age groups using the Population Estimates for Age-Related Physiology (PEAR) approach. The unbound fraction of KY0467 was 2.03% in infants and 1.98% in children, whereas the blood-to-plasma concentration ratio was 1.09 in both groups (Equations 1, 2). Each virtual pediatric population included 100 subjects: infants weighing 4.23–13.15 kg and children weighing 12.05–19.63 kg, with 50 females and 50 males in each group. The simulation duration was 48 h. Population simulations were conducted to determine doses in infants and children that achieved exposures comparable to those in adults administered 30 mg KY0467, as shown in Figures 4A,B. The predicted effective doses were 0.5  mg kg^-1^/day for infants and 9 mg/day for children. The corresponding exposures in both pediatric groups fell within the 90% prediction interval of the exposure observed in adults receiving the 30 mg dose.

**FIGURE 4:**
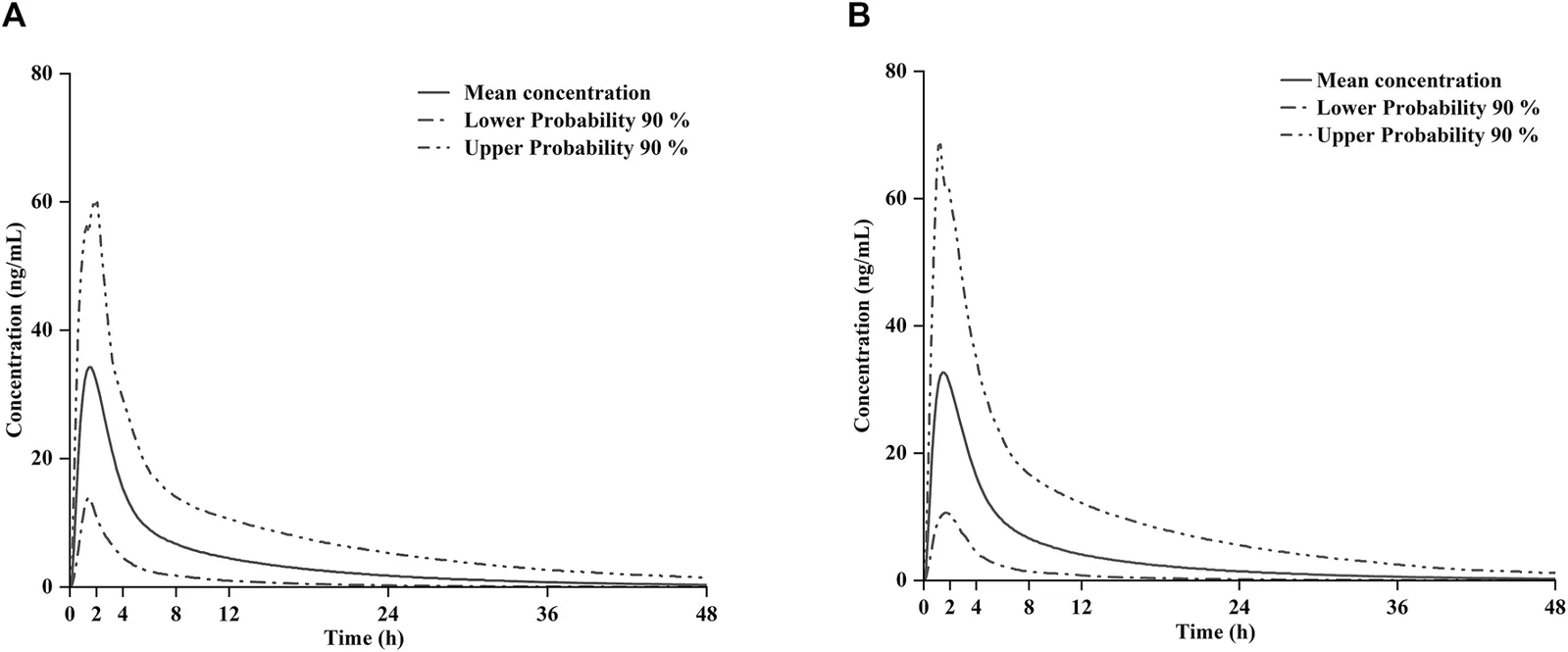
**(A)** Predicted plasma concentration versus time profiles of KY0467 after oral administration of 0.5 mg/kg/day in infants. **(B)** Predicted plasma concentration versus time profiles of KY0467 after oral administration of 9 mg/day in children.

## 4 Discussion

As a novel agent under clinical development for the treatment of hand, foot and mouth disease (HFMD) in pediatric population, the absorption, distribution, metabolism and excretion (ADME) properties of KY0467 remain poorly characterized. In this study, a mechanistic PBPK model for KY0467 was developed and validated in a stepwise manner:

First, the PBPK model was established by integrating physiological and drug-specific parameters, then optimized and verified using preclinical pharmacokinetic data from rats and beagles. Secend, several human clearance prediction methods were compared to forecast first-in-human pharmacokinetics of KY0467 in healthy Chinese adults, with subsequent validation using clinical trial data. Finally, the validated model was extrapolated to infants and children to support *a priori* pharmacokinetics predictions in these groups.

KY0467 is a basic compound with high permeability but low solubility, suggesting that its oral absorption is likely dissolution rate-limited. Following single intravenous administration, total plasma clearance (CL_p_) of KY0467 was 18.67 mL/min/kg in rats and 13.47 mL/min/kg/in beagles. Using a Rbp of 1.1 for both species, blood clearance (CL_b_) was estimated to be 16.97 and 12.25 mL/min/kg in rats and beagles, respectively. The hepatic extraction ratio (CL_b_/Q_H_) was calculated to be 31% in rats and 40% in beagles, indicating that KY0467 exhibits moderate clearance in both preclinical species.

The pharmacokinetic profile of KY0467 has been characterized in preclinical species. As detailed in Supplementary Table S1, KY0467 demonstrated rapid absorption (t_max_ 1–4 h) and favorable bioavailability in both rats and beagles. The drug exhibited a large volume of distribution and was primarily excreted via feces. Notably, systemic exposure showed non-linear kinetics, increasing in a greater-than-dose-proportional manner in rats but less-than-dose-proportional in beagles.

Moreover, accurate prediction of the rate and extent of absorption is critical for reliable oral pharmacokinetic simulations. In the present study, tissue distribution was described using a perfusion-limited model, and tissue-to-plasma partition coefficients (Kp) were estimated using the Lukacova method. The resulting predicted V_ss_ values corresponded closely with observed data in rats and beagles. By fixing CL and V_ss_ in the PBPK model, we were able to focus specifically on characterizing the absorption behavior of KY0467.

Notable discrepancies were observed between simulated and observed T_max_ values. Given that lipophilic weak bases with Log P > 2 and pKa>6.5 are prone to lysosomal trapping, we further explored the influence of this process on drug absorption in the PBPK model (Bolger MB et al., 2018; Wu DN et al., 2022; Samant TS et al., 2017). A reduced unbound fraction of drug in enterocytes (f_u,ent_) may slow the rate of drug transfer from enterocytes into the portal vein. To capture this mechanism, the enterocyte unbound fraction was optimized in beagle and human PBPK models. However, it should be acknowledged as a limitation that this optimization was based on model fitting to observed *in vivo* data rather than direct *in vitro* measurements of lysosomal trapping. While the adjusted f_u,ent_ values successfully captured the delayed absorption phase, the lack of specific *in vitro* verification for this parameter introduces a degree of uncertainty regarding the precise magnitude of intracellular retention.

In beagles, the enterocyte unbound fraction was adjusted to 1.3% to account for lysosomal accumulation and/or phospholipid binding of KY0467 in enterocytes, which affects the rate of appearance in plasma. This parameter was calibrated using the 10 mg/kg oral dataset and subsequently verified at 30 mg/kg and 90 mg/kg, yielding good agreement between simulated and observed concentration time profiles. In humans, the enterocyte unbound fraction was optimized to 5% to reflect the same mechanism. The human model was developed using the 10 mg clinical dataset and validated at 30 mg and 100 mg, demonstrating consistent predictive performance across all tested doses.

The pharmacokinetics of orally administered KY0467 in humans were predicted using available preclinical and *in vitro* data before initiation of the clinical trial. Human clearance was predicted using multiple methods, among which the TS_rat-beagle_ approach provided results most consistent with clinical observations. According to the literature, allometric scaling methods generally yield predictions within 2- to 3-fold error for most drugs (covering 80%–90% of compounds), except for those with a hepatic extraction ratio below 0.3 (Liu D et al., 2016).

Before the clinical trials, purely *a priori* predictions of human pharmacokinetics (PK) were indeed performed in the absence of human data. We utilized two distinct methods to estimate human clearance (CL). However, the clinical PK data across different dose levels exhibited non-linearity (Supplementary Table S1), suggesting saturable metabolism. On the basis of *in vitro* microsomal incubations, KY0467 was found to be predominantly metabolized by CYP3A4 (approximately 90%). To capture this non-linear behavior and improve the model’s predictive power, we incorporated Michaelis-Menten kinetics to describe the non-linearity.

To simulate the observed non-linear PK, we scaled the linear CL to derive the necessary enzymatic parameters. We fixed the apparent K_m_ which reflects intrinsic affinity and remains relatively constant and optimized V_max_ using clinical data, as it is more susceptible to physiological variations. The *in vitro* V_max_ and K_m_ values for CYP3A4 were integrated into the final adult PBPK model to characterize the nonlinear pharmacokinetic behavior via the in vitro–in vivo extrapolation (IVIVE) approach (Pierrillas PB et al., 2019; Abuasal BS et al., 2012; Snoeys J et al., 2015). Predicted AUC values of orally administered KY0467 were within two fold of the observed values, which is consistent with published criteria and regarded as acceptable accuracy for PBPK modeling (De Buck et al., 2007).

Additionally, target effective dose simulations were performed using virtual adult demographic data. The results indicated a 90% probability that observed values would fall within the simulated range. For population simulations of the 30 mg adult dose, the coefficients of variation (CV %) for CYP3A4 expression, gut first-pass extraction, and permeability were optimized to 40%,10% and 20%, respectively. These parameters were subsequently used to predict effective doses in infants and children, assuming target exposures similar to those in adults receiving the 30 mg dose. A sensitivity analysis was conducted to assess the influence of hepatic CYP3A4 expression level on the pharmacokinetics of KY0467 in infants. The results demonstrated a strong negative correlation between hepatic CYP3A4 expression and drug exposure (AUC). Variations in enzyme expression levels led to substantial changes in predicted AUC_t_, suggesting that the hepatic CYP3A4 expression level of CYP3A4 is a critical determinant of drug clearance in this population (Supplementary Figure S1). Although external validation of the KY0467 pediatric PBPK model was not feasible due to the absence of clinical pediatric data, confidence in the model’s predictive performance was gradually strengthened throughout the model development and verification process.

## 5 Conclusion

In conclusion, the PBPK model of KY0467 was successfully developed and validated in rats and beagles, and subsequently extrapolated to healthy adults. The validated adult model was further scaled to pediatric populations to predict drug exposure and estimate effective doses, thereby providing a valuable reference for the design of dosing regimens in pediatric clinical trials.

## Data Availability

The original contributions presented in the study are included in the article/Supplementary Material, further inquiries can be directed to the corresponding author.
