# Supplementary material for: Physiologically-based pharmacokinetic model to predict pharmacokinetics of KY0467 in humans and guide dose selection in infants and children
Source: Front Pharmacol. 2026 Oct 12;17:1866331. doi: 10.3389/fphar.2026.1866331 (PMC13700209; doi:10.3389/fphar.2026.1866331)
Supplement: Supplementary file 1 [file Table1.docx]

| Species | Dose | C_max_(ng/mL) | T_max_(h) | T_1/2_(h) | AUC_0-t_ (ng∙h/mL) | F% |
| --- | --- | --- | --- | --- | --- | --- |
| Rats | 15 mg/kg | 92.5  (32.0) | 1(0.00) | 2.86  (0.506) | 275.5  (92.0) | 6.1 |
|  | 50 mg/kg | 360.5  (172) | 0.833  (0.289) | 2.59  (0.0929) | 1436.65  (613) | 9.3 |
|  | 150 mg/kg | 2157.7  (1156) | 4.00  (0.00) | 2.76  (0.881) | 10815.21  (6080) | 22.4 |
| Beagles | 10 mg/kg | 656.4  (502) | 2.33  (1.53 ) | 3.87  (0.577 ) | 3583.5  (3718 ) | 30.8 |
|  | 30 mg/kg | 2674.2  (442) | 1.67  (0.577) | 3.53  (0.291) | 11460.9  (4492) | 32.8 |
|  | 90 mg/kg | 2405.2  (963) | 3.00  (1.73) | 4.41  (1.17) | 13774.6  (7102) | 13.2 |
| Humans | 10 mg | 5.760  (4.97) | 1.3  (0.58) | 7.45  (5.88) | 30.26  (27.8) | / |
|  | 30 mg | 22.675  (6.79) | 2.1  (0.83) | 8.93  (2.79) | 158.88  (42.4) | / |
|  | 100mg | 74.450  (40.19) | 2.3  (0.71) | 13.7  (5.05) | 741.99  (272.76) | / |

Supplementary Table S1.The pharmacokinetic data of KY0467 in rats, beagles and humans. (Mean±SD)
